# Supplementary material for: Histone variant H2A.Z mutant suppresses the senescence-associated secretory phenotype
Source: bioRxiv. 2026 Apr 13:2026.02.16.706244. Originally published 2026 Feb 17. Preprint. [Version 2] doi: 10.64898/2026.02.16.706244 (PMC12934968; doi:10.64898/2026.02.16.706244)

Supplementary Table 1
List of Sybr green primers used for qPCR

Gene Name	Forward and Reverse PCR primer sequence
ICAM1	GGTTGAACCCCACAGTCACCTATG<br>GCTGTAGATGGTCACTGTCTGCAG
IL1A	AGTGCTGCTGAAGGAGATGCCTGA<br>CCCCTGCCAAGCACACCCAGTA
IL1B	TGCACGCTCCGGGACTCACA<br>CATGGAGAACACCACTTGTTGCTCC
IL6	AAATTCGGTACATCCTCGACG<br>TTTCACCAGGCAAGTCTCC
IL8	GAGTGGACCACACTGCGCCA<br>TCCACAACCCTCTGCACCCAGT
MMP1	ATCGGCCCACAAACCCCAAA<br>TGGCAGTTGTGGCCAGAAAACA
ACTB	AGAGCTACGAGCTGCCTGAC<br>AGCACTGTGTTGGCGTACAG
GAPDH	GAAGGTGAAGGTCGGAGTC<br>TTGAGGTCAATGAAGGGG
CDKN2A	CTGCCCAACGCACCGAATAG<br>CCACCAGCGTGTCCAGGAAG
CDKN1A	CTGCAGGGGACAGCAGAGG<br>CCGGCGTTTGGAGTGGTAG
HA-H2A.Z	ATGGCCTACCCCTACGACGTG<br>AACCGCCTTTGTCTTGGCCTTTC
H2AZ1	GGTCCGATTAGCCTTTTCTCTG<br>AACCGCCTTTGTCTTGGCCTTTC

Supplementary Figure 1

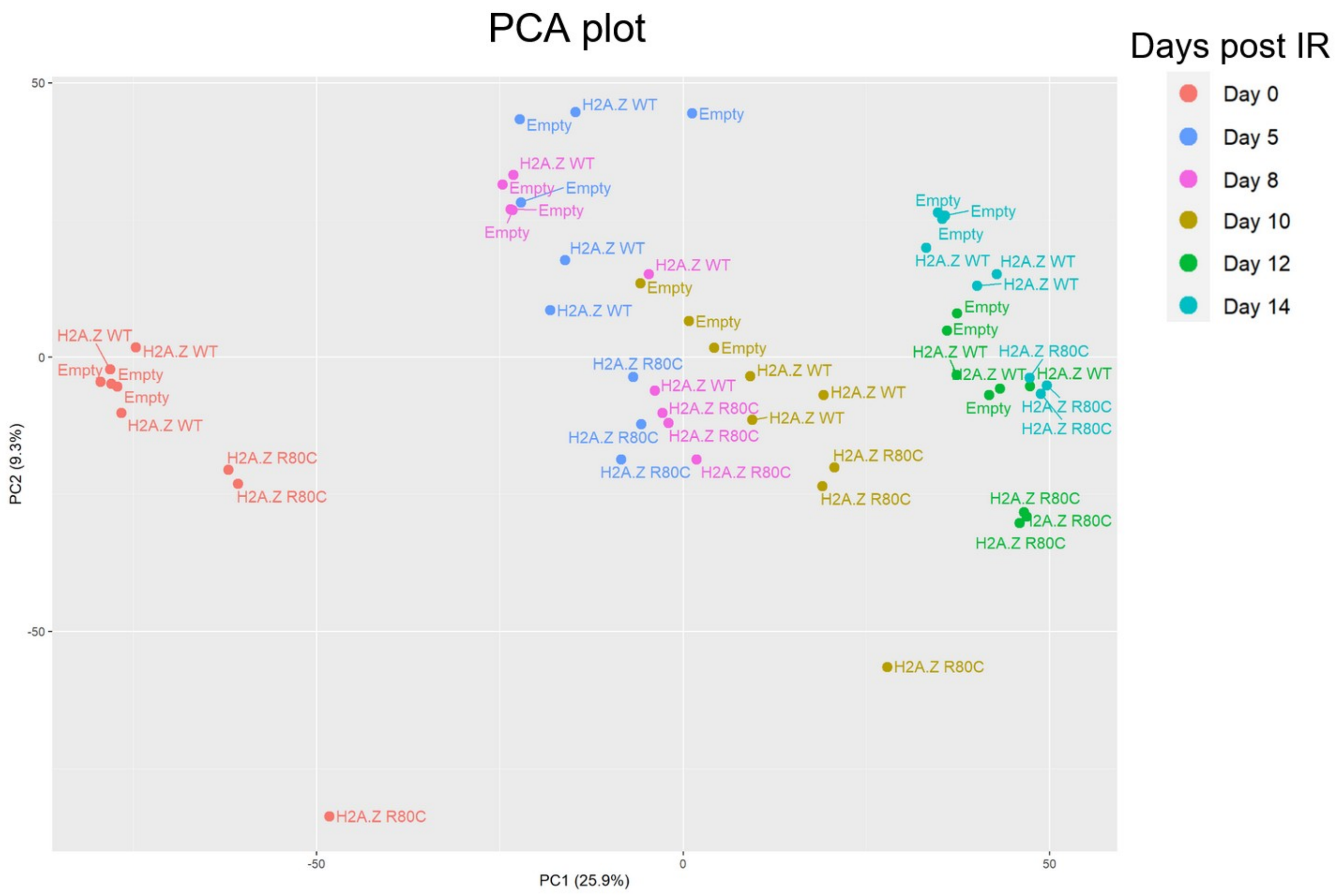

PCA plot showing PC1 and PC2 of bulk RNA-seq samples colored based on days elapsed post irradiation, including percentage of variances explained by the respective primary component

Supplementary Figure 2

Heatmap of differentially expressed genes in cluster 1

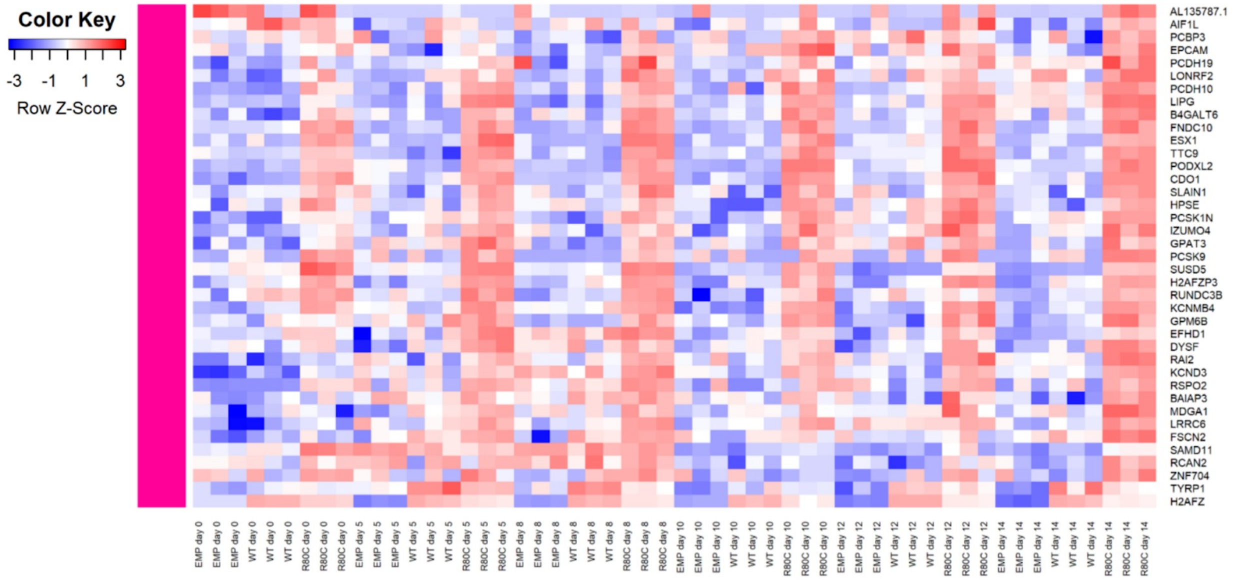

Functional enrichment analysis of cluster 1

id	source	term_id	term_name	term_size		p_value
1	KEGG	KEGG:00561	Glycerolipid metabolism	61		4.2e-02
2	KEGG	KEGG:04979	Cholesterol metabolism	50		4.2e-02
3	WP	WP:WP4522	Metabolic pathway of LDL, HDL and TG, including diseases	17		3.4e-03

g:Profiler (biit.cs.ut.ee/gprofiler)

Heatmap of differentially expressed genes in cluster 2

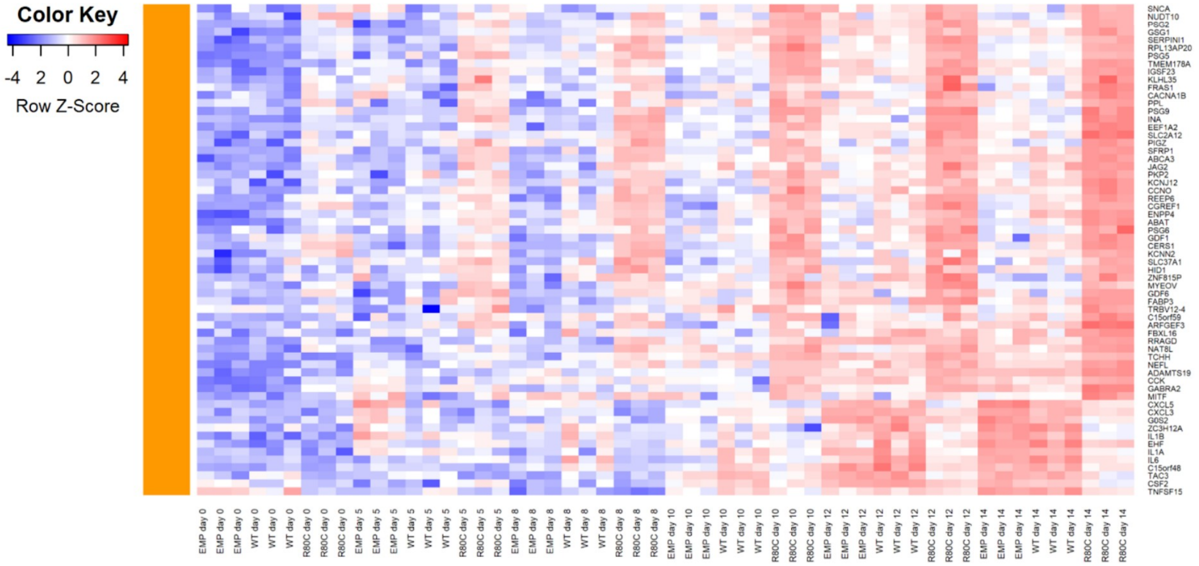

Functional enrichment analysis of cluster 2

id	source	term_id	term_name	term_size		p_value
1	GO:MF	GO:0005125	cytokine activity	235		5.1e-06
2	GO:MF	GO:0030546	signaling receptor activator activity	502		1.2e-05
3	KEGG	KEGG:04060	Cytokine-cytokine receptor interaction	293		5.9e-05
4	WP	WP:WP530	Cytokines and inflammatory response	26		3.7e-04
5	WP	WP:WP5095	Overview of proinflammatory and profibrotic mediators	127		4.2e-04

g:Profiler (biit.cs.ut.ee/gprofiler)

Heatmap of differentially expressed genes in cluster 3

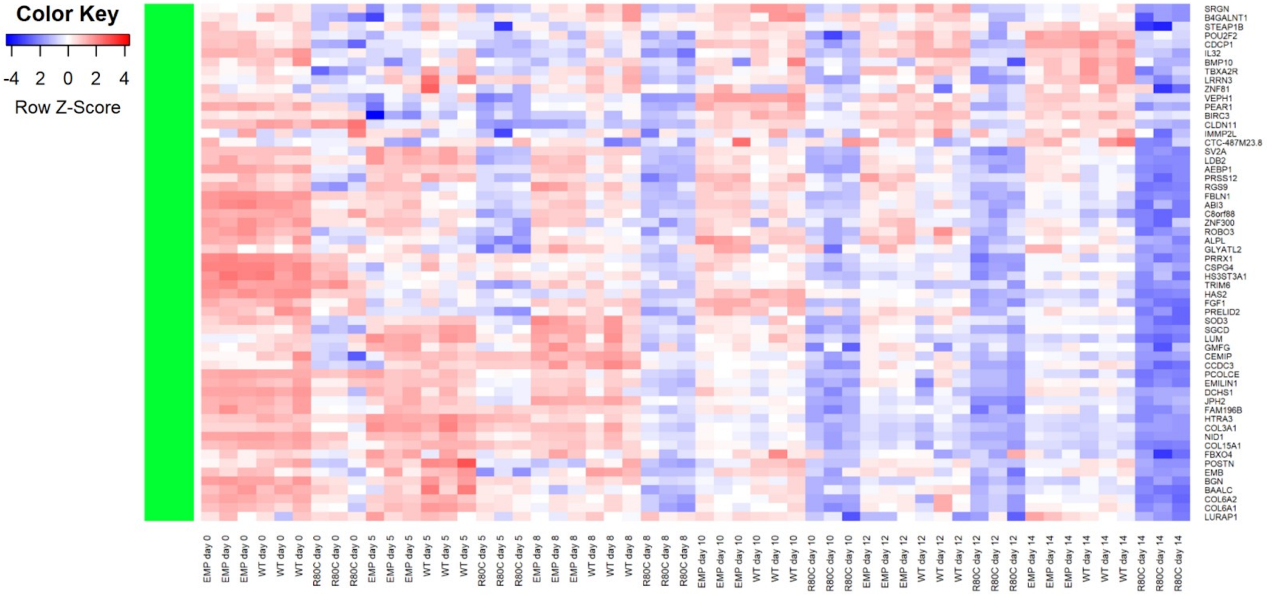

Functional enrichment analysis of cluster 3

id	source	term_id	term_name	term_size		p_value
1	GO:MF	GO:0005201	extracellular matrix structural constituent	173		5.8e-12
2	GO:MF	GO:0005518	collagen binding	69		3.1e-06
3	GO:CC	GO:0031012	extracellular matrix	560		3.6e-12
4	REAC	REAC:R-HSA-1474244	Extracellular matrix organization	298		1.5e-07
5	HPA	HPA:0461432	skin 1; extracellular matrix[≥Medium]	48		6.6e-12

g:Profiler (biit.cs.ut.ee/gprofiler)

Heatmap of differentially expressed genes in cluster 4

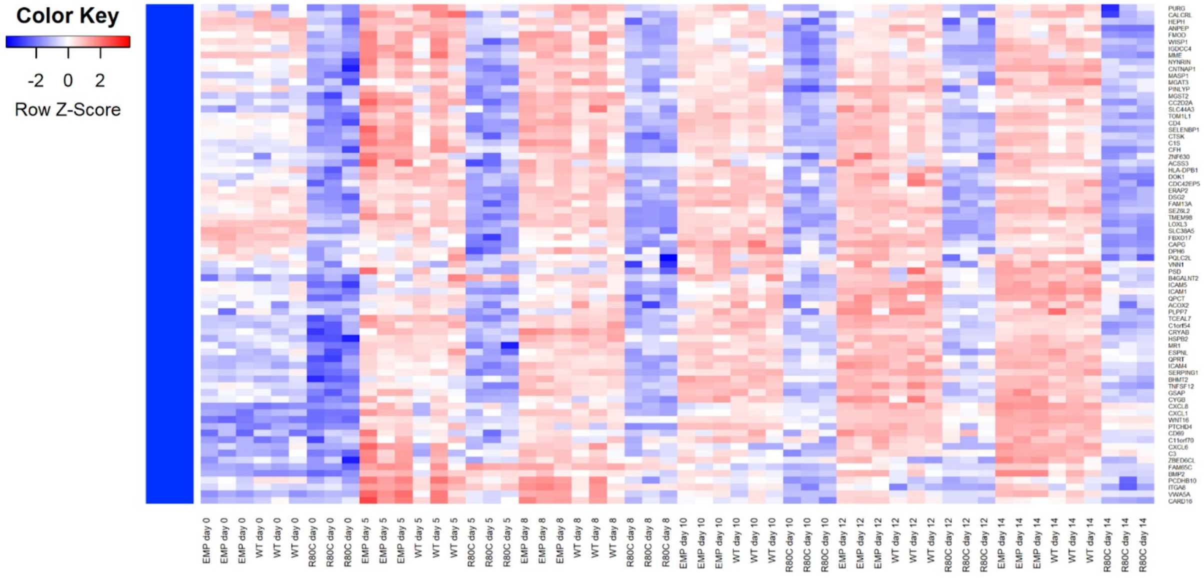

Functional enrichment analysis of cluster 4

id	source	term_id	term_name	term_size		p_value
1	GO:CC	GO:0005576	extracellular region	4303		2.6e-04
2	GO:CC	GO:0009986	cell surface	908		4.2e-04
3	WP	WP:WP2806	Complement system	99		3.6e-04

g:Profiler (biit.cs.ut.ee/gprofiler)

Supplementary Figure 3

Local network cluster (STRING)

Cluster	Count in Network	Strength	False Discovery Rate	Description
CL:18273	6 of 34	1.38	0.00047	Chemokine receptor binding
CL:16433	6 of 57	1.16	0.0032	Collagen biosynthesis and modifying enzymes
CL:16569	6 of 58	1.15	0.0032	Mixed, incl. elastic fibre formation and MMP

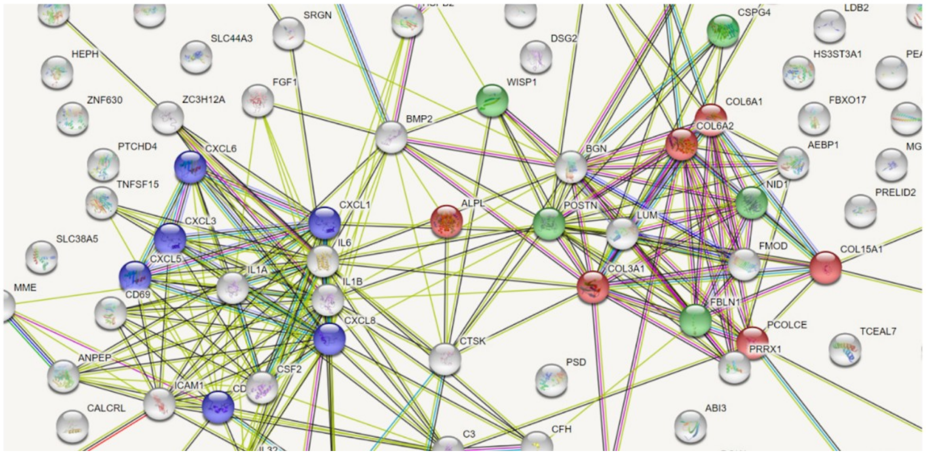

Supplementary Figure 4

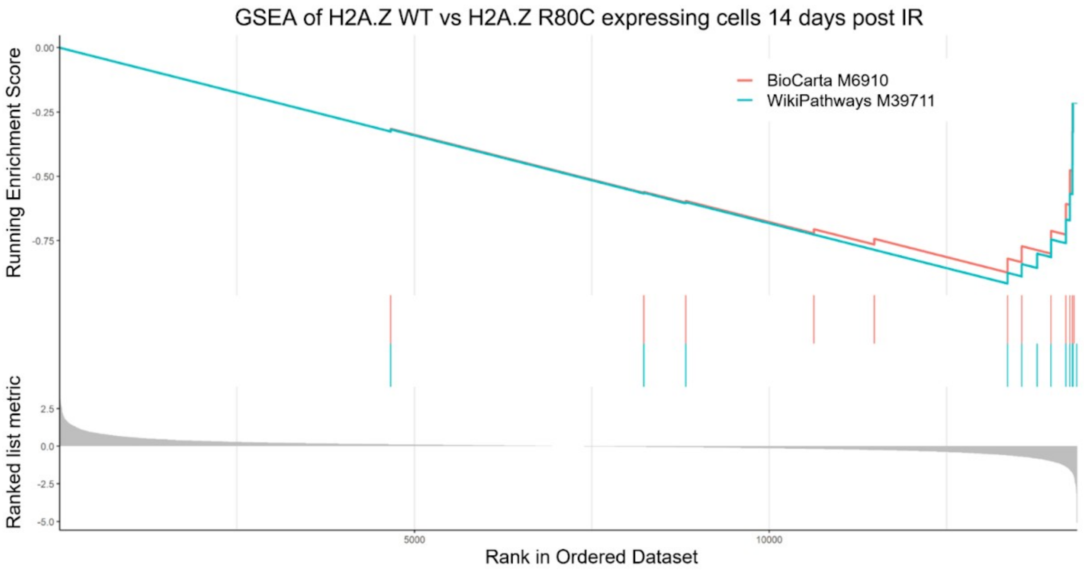

(A) STRING database analysis of genes enriched in H2A.Z WT expressing cells compared to H2A.Z R80C expressing cells at 14 days post irradiation (B) Gene Set Enrichment Analysis shows that genes associated with Cytokines and Inflammatory Response (BioCarta M6910 (BIOCARTA_INFLAM_PATHWAY) and WikiPathways M39711 (WP_CYTOKINES_AND_INFLAMMATORY_RESPONSE)) are strongly enriched in H2A.Z WT expressing cells compared to H2A.Z R80C expressing cells. BioCarta M6910 has an enrichment score of -0.87 and WikiPathways M39711 an enrichment score of -0.92.

Supplementary Figure 5

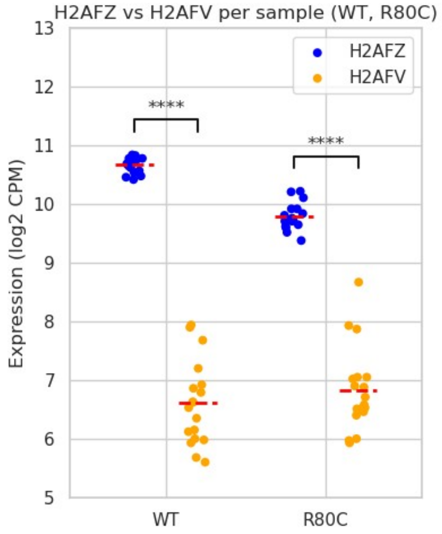

Expression (log2 CPM) of H2AFV and H2AFZ as observed in H2A.Z WT and H2A.Z R80C expressing cells from days 0 to 14 during senescence induction.

Supplementary Figure 6

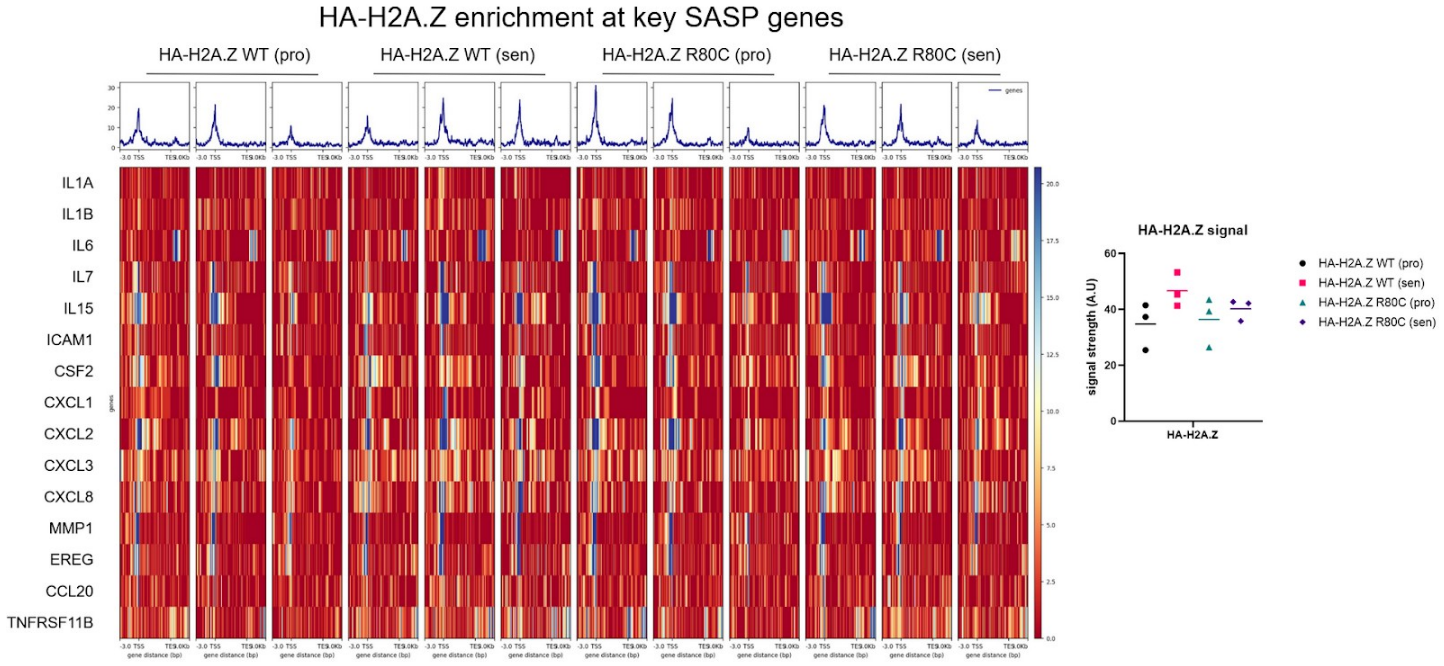

Supplement: Supplement 1 [file media-1.pdf]
